# Single Incision Laparoscopic Cholecystectomy for Gallbladder Duplication

**DOI:** 10.1155/2015/589313

**Published:** 2015-07-22

**Authors:** Esin Kabul Gürbulak, Hamdi Özşahin, Yiğit Düzköylü, Ismail Ethem Akgün, Muharrem Battal, Bünyamin Gürbulak

**Affiliations:** ^1^Department of General Surgery, Şişli Hamidiye Etfal Training and Research Hospital, Şişli, 34371 Istanbul, Turkey; ^2^Department of General Surgery, Istanbul Training and Research Hospital, Fatih, 34098 Istanbul, Turkey

## Abstract

Duplication of the gallbladder is a rare congenital anomaly of the gallbladder, with an estimated prevalence of 1–3 per 3800 individuals. Unless properly diagnosed preoperatively, it can lead to biliary tract injuries and postoperative complications which may require reoperative surgeries. While previously reported cases have been treated with conventional laparoscopic cholecystectomy (LC), treatment with single incision laparoscopic surgery (SILS) has not been reported yet. We herein present the case of a 58-year-old female with gallbladder duplication who was successfully treated with SILS cholecystectomy.

## 1. Introduction

Duplication of the gallbladder is a very rare congenital entity seen in 1–3 patients among every 10.000 patients diagnosed with gallbladder anomalies [1, 2]. Appropriate preoperative diagnosis is necessary to help avoid potential complications and/or recurrent surgical procedures [3].

The gold standard treatment for benign gallbladder disorders is laparoscopic cholecystectomy (LC). Recent developments in laparoscopic techniques have allowed the procedure to be performed safely through a single incision. However, to date, only 20 cases of gallbladder duplication have been reported and all cases were managed via the conventional LC [4]. The management of gallbladder duplication via a single laparoscopic incision has not been reported yet. The aim of this report is to describe the safety and feasibility of SILS for the management of duplication of the gallbladder in a female patient.

## 2. Case Description

**Figure 1:**
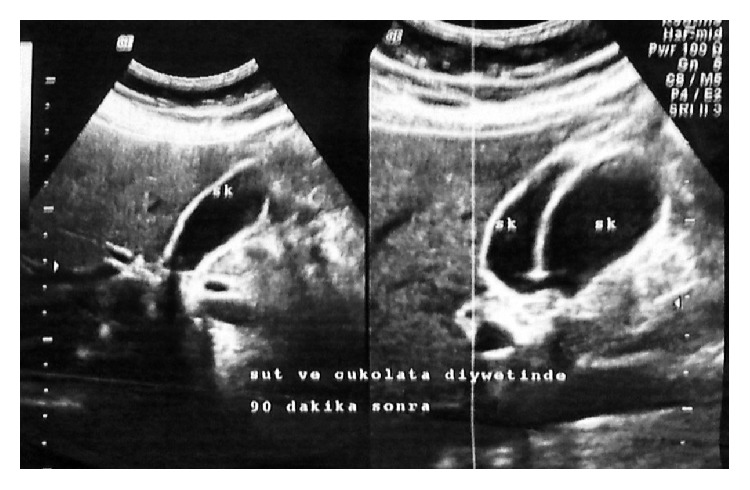
Ultrasound image of both gallbladders.

**Figure 2:**
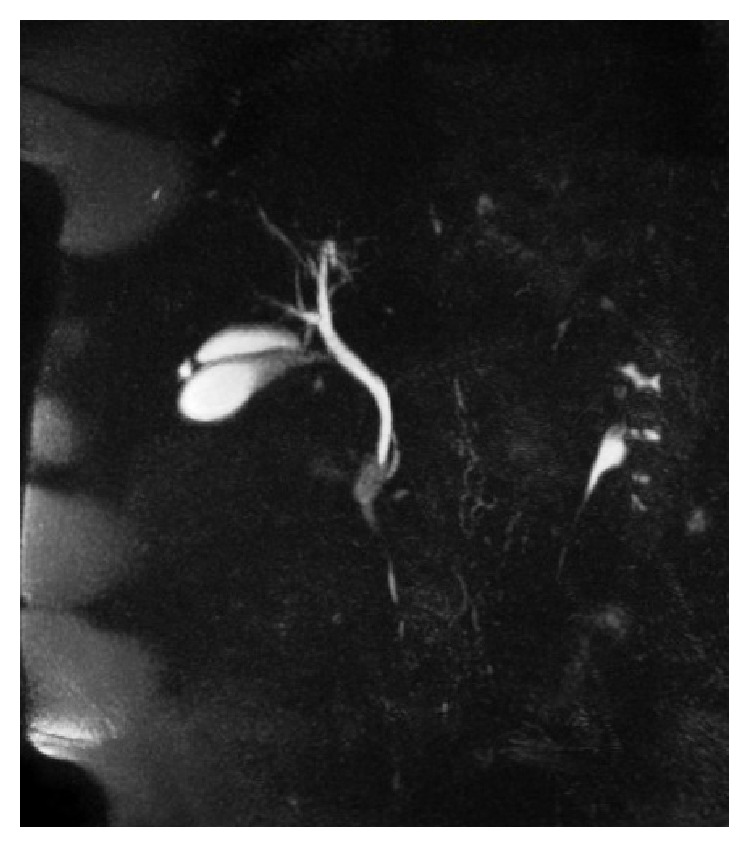
MRCP image showing the single cystic duct, draining to a common hepatic duct.

A 58-year-old Caucasian female was admitted to our general surgery department, with complaints of dyspepsia and intermittent epigastric pain. Except for right upper quadrant pain and tenderness that was present on palpation, all other physical examination findings were normal. Laboratory results including liver function tests were found to be within normal limits. Upper abdominal ultrasound imaging revealed 2 separate gallbladders lying side by side (Figure 1). In one of the gallbladders, dense bile micelle and a few calculi were seen. The relationship between the gallbladders and the cystic duct was nonconclusive on ultrasound imaging. Magnetic resonance imaging cholangiopancreatography (MRCP) imaging was performed to better clarify the anatomical relationship. On MRCP imaging two separate gallbladders that were independent of each other in parts of the fundus and corpus-body were demonstrated. There was adherence to one another in the neck, draining to a common bile duct through a single cystic duct (Figure 2). The common bile duct, hepatic duct, and intrahepatic bile ducts were found to be in normal calibre and anatomic structure. In order to accurately determine the anatomy of the whole biliary system, cholecystectomy was planned for treatment. A single incision laparoscopic cholecystectomy procedure was performed per surgeon's preference.

### 2.1. Operative Procedure

**Figure 3:**
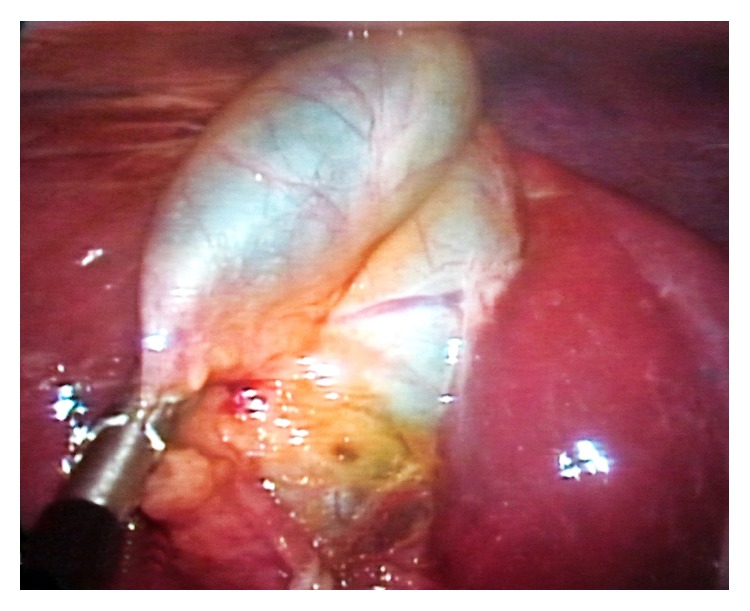
Laparoscopic image of duplicated gallbladder.

**Figure 4:**
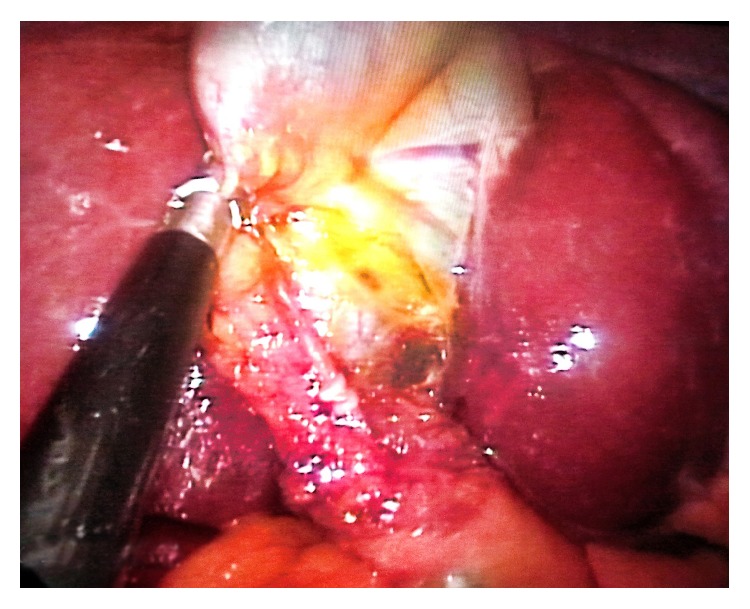
Single cystic duct, in front of dissected cystic artery, following the dissection of Calot.

**Figure 5:**
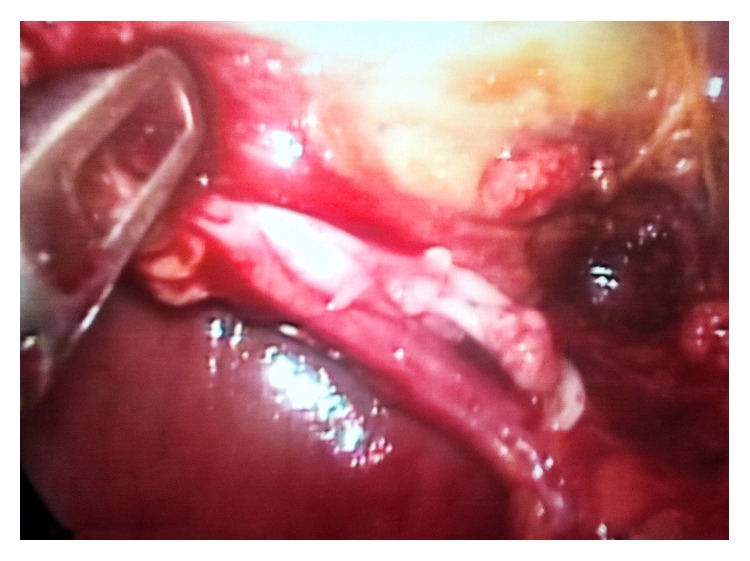
Clipped cystic duct.

Following a 2-cm incision, a SILS port with three 5-mm ports (Covidien, Inc., Norwalk, CT, USA) was placed through the umbilicus. Two separate gallbladders were observed at laparoscopic exploration, using a camera of 5-mm and 30° (Figure 3). By using a laparoscopic grasper, the Hartmann pouch was hung and surrounding tissues were dissected beginning from the neck with a laparoscopic dissector. After dissection of the triangle of Calot, 2 separate gallbladders were identified. Both gallbladders were fused distally, forming a common neck segment. The structure of a single cystic duct appeared (Figures 4 and 5). The cystic artery was found to originate from the right branch of hepatic artery as a single structure and moved on to the neck solely. An intraoperative cholangiography was not performed because a single cystic duct that was confirmed on preoperative radiologic imaging was clearly visible intraoperatively. Then both the cystic duct and artery were clipped and dissected safely. Two gallbladders were dissected from the liver bed in a retrograde fashion and resected en bloc from the umbilicus with the SILS port. The procedure was completed in duration of 48 minutes with no intraoperative complications encountered. The patient was discharged from the hospital on postoperative day one. Histopathologic evaluation of the dissected gallbladders revealed chronic cholecystitis, with pyloric metaplasia on the mucosa of one of the gallbladders.

## 3. Discussion

Although congenital anomalies of the biliary system are frequent, gallbladder duplication is a relatively uncommon anomaly. In a case series, published by Harlaftis et al. in 1977, 207 duplicated and 8 triple gallbladders were reported. Accordingly, anatomic variations of gallbladder duplications were classified based on the location and number of cystic ducts. Type 1 includes gallbladder duplication separated with a septum and two separate gallbladders that fuse in the neck to form a single cystic duct; type 2 includes accessory gallbladders with two different cystic ducts [5].

The symptoms and signs of gallbladder duplication are similar to those of patients with a single gallbladder. There is no scientific evidence to associate the anomaly with increased cholecystitis or malignancy. When diagnosed incidentally, prophylactic cholecystectomy is not required [5]. Nevertheless, appropriate diagnosis is necessary to avoid biliary tract injuries that can occur during surgeries, postoperative complications, or a need for a reoperative procedure. Despite the known value of preoperative diagnosis, no imaging technique has been accepted as a gold standard modality yet. Ultrasound is usually the initial imaging choice, as it can show the duplicated gallbladder structure in cases where the gallbladders are located separately in the liver bed. However, specific classification of duplication can be made only if two cystic ducts are seen thus making it challenging to observe a cystic duct. Unless dilated, determining the type of the duplication with ultrasound is not feasible [6, 7]. Another noninvasive imaging technique is the magnetic retrograde cholangiopancreatography (MRCP). Similar to the other invasive techniques, such as endoscopic retrograde cholangiopancreatography (ERCP) and percutaneous transhepatic cholangiography (PTC), MRCP has been reported to be effective in diagnosing biliary disorders [8]. In our case, ultrasound imaging was useful in identifying the duplication, but MRCP was needed to determine the anatomy of the biliary system in more detail. The anomaly in this case was described as type 1 duplication according to Harlaftis' classification.

Similar to other biliary disorders, surgical resection of the duplicated gallbladders is warranted in symptomatic patients [9]. Resection of both gallbladders is necessary to avoid cholecystitis of the remaining gallbladder or a need for reoperative surgery [9, 10]. Considering the risk of injury that might occur, Kim et al. [11] previously recommended open surgery for gallbladder duplications. Currently, laparoscopic cholecystectomy (LC) is accepted as the gold standard approach in gallbladder surgery. In a recent case report and review of the literature, Walbot described 21 gallbladder duplications that were successfully treated with LC [12].

With increased surgical experience and technical improvements in laparoscopy, SILS has been introduced. It is accepted as a less invasive alternative to conventional laparoscopic surgery. Numerous studies have shown single incision laparoscopic cholecystectomy to be an applicable, safe, and effective surgical method. Advocates have recommended it for noncomplicated gallbladder diseases [13–17] due to other advantages such as less postoperative pain, less need for analgesic agents, and better cosmetic results. The procedure is however limited by its poor ergonomy considering the insertion of multiple devices (laparoscope and other devices) via a single incision. This can make the procedure harder to perform compared to conventional LC [16]. Another challenging aspect of SILS is the poor communication between the camera assistants and the surgeon [17].

Over the past 5 years the SILS technique has been successfully incorporated into the treatment algorithm for noncomplicated biliary disorders at our center. With our experience extending beyond the learning curve, we attempted SILS for the treatment of a duplicated gallbladder case. To the best of our knowledge this is the first report describing SILS for duplicated gallbladder in the medical literature. We believe that, with appropriate preoperative imaging diagnosis, it is feasible and safe to perform this technique as conventional LC in noncomplicated gallbladder duplications.
